# Fused Spinopelvic Angle in Adult Spinal Deformity with Pure Sagittal Malalignment: A Postoperative Threshold Associated with Proximal Junctional Kyphosis

**DOI:** 10.3390/jcm15187055

**Published:** 2026-09-11

**Authors:** Ki Young Lee, Jung-Hee Lee, Kyung-Chung Kang, Hong-Sik Park, Woo-Jae Jang, Eugene J. Park

**Affiliations:** 1 Department of Orthopedic Surgery, Graduate School, College of Medicine, Kyung Hee University, 23 Kyungheedae-ro, Dongdaemun-gu, Seoul 02447, Republic of Korea; keyng39@hanmail.net (K.Y.L.); futurespine@gmail.com (K.-C.K.); maalouf@hanmail.net (H.-S.P.); rapture138@naver.com (W.-J.J.); 2 Spine Center, Bogang Hospital, Daegu 42801, Republic of Korea; pjj841229@gmail.com

**Keywords:** adult spinal deformity, lumbar lordosis, pelvic incidence, proximal junctional kyphosis, sagittal imbalance, spinopelvic alignment

## Abstract

**Background:** Determining the appropriate magnitude of lumbar lordosis (LL) correction in adult spinal deformity (ASD) remains controversial. The fused spinopelvic angle (FSPA), a posture-independent parameter, may provide a geometric measure of fused-construct orientation associated with proximal junctional kyphosis (PJK) risk. **Methods:** This retrospective single-center study included 258 patients aged ≥65 years with ASD and pure sagittal malalignment associated with lumbar degenerative kyphosis/drop body syndrome who underwent long-segment fixation from T10 to the sacrum with sacropelvic fixation. Patients were divided into non-PJK (*n* = 135) and PJK (*n* = 123) groups. Adjusted nested logistic regression models, receiver operating characteristic (ROC) analysis, and DeLong comparison were used to evaluate the association of FSPA with PJK and its incremental value beyond postoperative pelvic incidence–lumbar lordosis (PI-LL). **Results:** Postoperative FSPA was lower in the PJK group (*p* < 0.001) and remained independently associated with PJK in the fully adjusted model including postoperative PI-LL (adjusted OR = 0.910 per degree; 95% CI, 0.866–0.957; *p* < 0.001). FSPA showed greater discriminatory ability than postoperative PI-LL (AUC, 0.694 vs. 0.596; difference, 0.099; 95% CI, 0.035–0.162; DeLong *p* = 0.002). Adding FSPA to the clinical model containing postoperative PI-LL significantly improved model fit (likelihood-ratio *p* < 0.001). The cohort-derived FSPA threshold of 2.38° yielded 64.2% sensitivity and 63.7% specificity. **Conclusions:** Higher postoperative FSPA was independently associated with lower odds of radiographic PJK and provided additional predictive information beyond postoperative PI-LL. The identified threshold of 2.38° may serve as a preliminary reference for postoperative alignment in this selected cohort.

## 1. Introduction

The prevalence of adult spinal deformity (ASD) has increased with prolonged life expectancy [1]. The primary goals of ASD surgery are to restore sagittal balance and obtain solid arthrodesis [2]. However, elderly patients with ASD frequently present with frailty, poor bone quality, and extensive degenerative changes [3,4], which predispose them to postoperative sagittal decompensation and inferior clinical outcomes [4,5,6]. Therefore, determining the appropriate degree of correction in elderly patients with severe sagittal malalignment remains a complex and multifactorial challenge for surgeons.

Recently, an age-specific alignment concept has been proposed, suggesting that undercorrection may be acceptable in elderly individuals, as anterior trunk shift and pelvic retroversion naturally increase with aging [7]. Proportional alignment concepts, including the global alignment and proportion (GAP) score, similarly evaluate alignment relative to individual pelvic morphology rather than relying on a single universal pelvic incidence-lumbar lordosis (PI-LL) target [8]. However, undercorrection has been associated with suboptimal clinical outcomes at ≥2-year follow-up [5], whereas excessive correction may increase the risk of mechanical complications, such as proximal junctional kyphosis (PJK) [9,10,11]. Accordingly, correction planning requires a balance between adequate restoration and avoidance of excessive correction in the context of each patient’s baseline deformity and the altered pathologic nature before surgery.

Surgical treatment of ASD commonly requires long-segment fixation extending distally to S1. In such cases, the spine becomes rigidly fixed from the uppermost instrumented vertebra (UIV) to the pelvis, creating a single fused construct. Because the fused segment is only mobilized from the hip joint, the fused spinopelvic angle (FSPA), a novel fixed parameter defined between the UIV and pelvis, has been introduced. As FSPA decreases, the UIV shifts posteriorly relative to the pelvis, increasing the risk of PJK [12]. Because FSPA directly reflects the geometric relationship between the UIV and pelvis and is relatively independent of compensatory posture, FSPA may complement established concepts. Similar to PI, recently introduced fixed angular parameters such as the T1 pelvic angle (T1PA) and L1 pelvic angle (L1PA) have also been used to assess sagittal alignment while minimizing the influence of compensatory mechanisms [13,14].

In the present study, we focused on a homogeneous cohort of patients aged ≥65 years with pure sagittal malalignment with lumbar degenerative kyphosis (LDK), redefined as “drop body syndrome (DBS)” by Yagi et al. [15]. Using FSPA as a key parameter [12], we aimed to investigate the association between postoperative fused construct alignment and PJK and to identify a cohort-derived FSPA threshold associated with PJK. Given the observational nature of the study, our aim was to characterize this radiographic association rather than to establish a causal relationship or a universally applicable surgical target.

## 2. Materials and Methods

### 2.1. Study Design and Patient Selection

The study was approved by the Institutional Review Board of Kyung Hee University Hospital (KHUH IRB 2021-10-080; approval date, 5 November 2021). The requirement for informed consent was waived due to the retrospective nature of the study.

We reviewed 335 consecutive patients aged ≥65 years who underwent deformity correction for ASD between 2008 and 2020. The inclusion criteria were as follows: First, severe sagittal malalignment satisfying all three radiographic criteria (sagittal vertical axis [SVA] > 50 mm, PI-LL mismatch > 10°, and pelvic tilt [PT] > 25°), with a minimum follow-up of 2 years after deformity correction. Second, long-segment fixation with sacropelvic fixation and setting of the uppermost and lowermost instrumented vertebrae at the T10 and S1 levels, respectively. Third, pure sagittal malalignment associated with LDK/DBS, characterized by clearly evident atrophy of the back musculature on axial magnetic resonance imaging or computed tomography (CT) images together with characteristic clinical features of LDK, including walking difficulty with progressive stooping, inability to lift heavy objects in front of the body, difficulty in climbing slopes, and the need for elbow support during standing activities, occasionally resulting in callus formation over the extensor surface of the elbow, as previously described [3].

Patients who did not meet these predefined criteria, including those with a UIV other than T10 or insufficient follow-up, were excluded. A history of previous spinal surgery was not considered an exclusion criterion if the patient otherwise fulfilled the predefined inclusion criteria. In the present study, “pure sagittal malalignment” was defined as a deformity phenotype in which sagittal malalignment attributable to LDK/DBS was the predominant deformity and the primary indication for deformity correction. Concomitant degenerative coronal changes were not considered exclusionary when sagittal malalignment remained the dominant deformity.

Patients were divided into PJK and non-PJK groups based on the presence of PJK during follow-up.

### 2.2. Radiographic Measurement

Sagittal alignment was evaluated using lateral 14 × 36-inch full spine radiographs obtained with patients standing in an unsupported neutral position with “fists-on-clavicle” [16]. Postoperative radiographic parameters were measured 1 month postoperatively. All digital radiographs were analyzed using validated software (Surgimap, version 2.3.2.1; Nemaris Inc., New York, NY, USA). Radiographs were independently measured by three experienced spine surgeons who were blinded to the patient’s PJK status at the time of measurement, and the mean of the three measurements was used for analysis. We evaluated PI, sacral slope (SS), PT, thoracic kyphosis (TK), thoracolumbar junctional angle (TL), LL, lumbosacral junctional angle (LS), and SVA. Sagittal Cobb angles were measured for TK (T5-12), TL (T10-L2), LL (T12-S1), and LS (L4-S1). PI, PT, and SS were measured using a standing lateral radiograph of the pelvis according to a previously described method [17].

**Figure 1:**
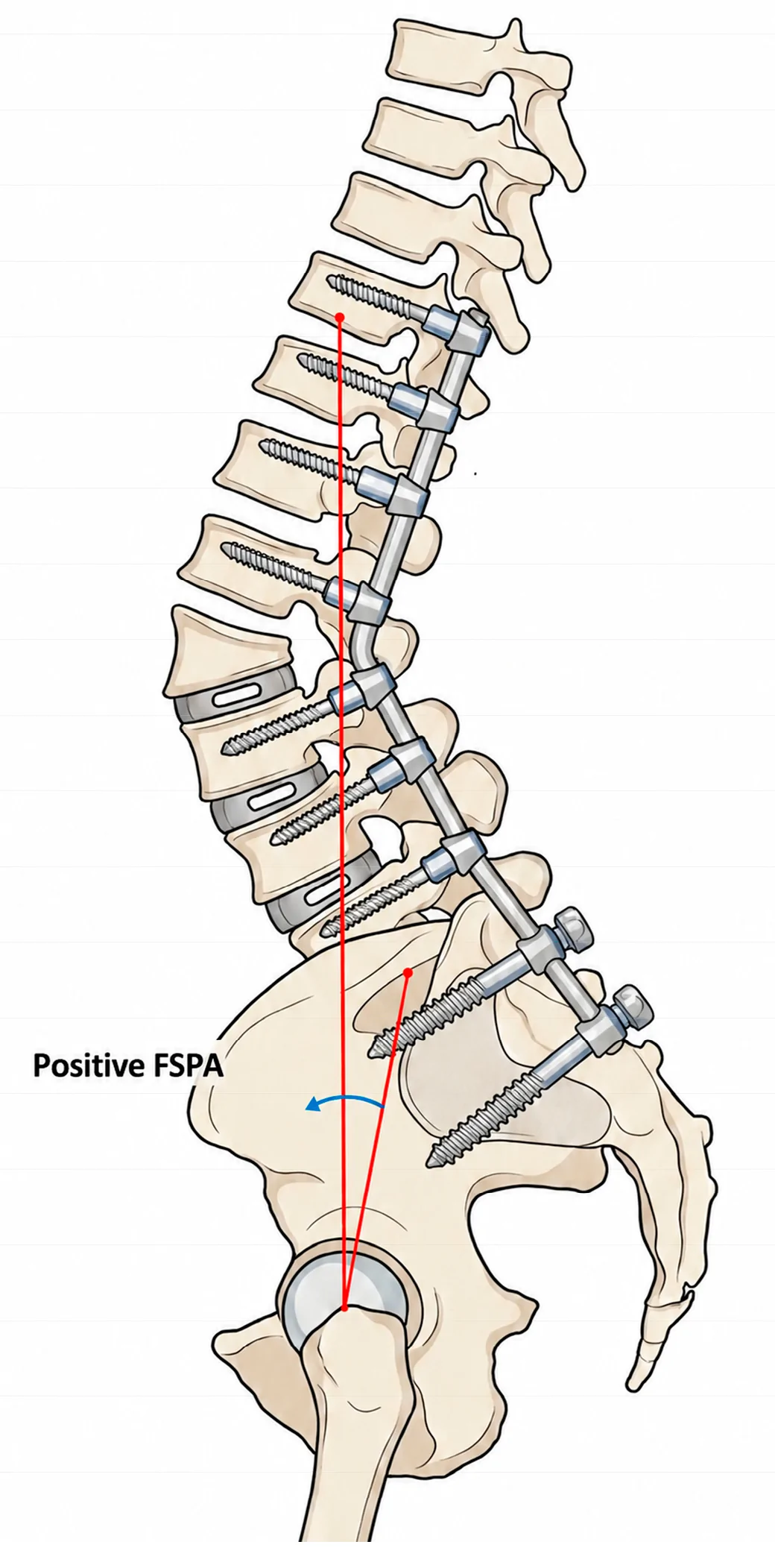
Schematic illustration of FSPA measurement. FSPA was defined as the angle between the lines connecting the hip axis to the center of the lower endplate of the upper instrumented vertebra and to the center of the S1 upper endplate. A positive value indicates that the UIV-based line is located anterior to the S1-based reference line.

Additional parameters included PI-LL mismatch, lordosis distribution index (LDI; L4-S1/LL × 100) [18], apex-LDI (LS/LL × 100) [18,19], and FSPA [12]. FSPA was defined as the angle between a line connecting the center of the inferior endplate of the UIV to the midpoint of the bicoxofemoral axis, and a line connecting the center of the sacral endplate to the same midpoint. FSPA was considered positive when the line passing through the UIV was located anterior to the sacral reference line. A schematic of the FSPA measurement is provided in Figure 1.

The proximal junctional angle (PJA) was measured from the inferior endplate of the UIV to the superior endplate of two vertebrae above the UIV. PJK was defined in accordance with the following criteria: proximal junction sagittal Cobb angle of ≥10° with a change in angle of at least 10° greater than the preoperative measurement [20].

Diagnosis of RF was based on rod breakage with a recent fusion mass fracture observed on plain radiography and CT, confirmed by increased uptake on either bone scan or bone single-photon emission computed tomography [21].

### 2.3. Clinical and Surgical Variables

Potential risk factors for PJK included patient factors (age, bone mineral density [BMD, g/cm^2^], body mass index [BMI, kg/m^2^], and preoperative degenerative lumbar scoliosis [DLS]), radiographic spinopelvic parameters (preoperative, postoperative, and last follow-up evaluations), and surgical factors (L5-S1 anterior lumbar interbody fusion [ALIF] vs. posterior lumbar interbody fusion [PLIF], correction method [pedicle subtraction osteotomy vs. lateral lumbar interbody fusion with posterior column osteotomy], and the use of accessory rod technique). All deformity correction procedures were performed at a single institution by the same surgical team, with all surgeries performed by the same senior spine surgeon. Although surgical techniques and minor aspects of perioperative management evolved during the study period, there was no predefined major transition in the overall treatment protocol that allowed the cohort to be meaningfully divided into distinct treatment eras. Preoperative BMD was measured using dual-energy X-ray absorptiometry (DXA) at the lumbar spine (L1–L4) and proximal femur, including the femoral neck and total hip. Examinations were performed using GE Lunar densitometry systems, including Lunar Prodigy Advanced and Lunar iDXA scanners (GE Healthcare, Madison, WI, USA); scanner software was updated during the study period. The BMD value corresponding to the skeletal site with the lowest T-score was used in the analysis. Patients with low BMD or osteoporosis received appropriate pharmacologic treatment according to their clinical condition, including injectable osteoporosis therapy when indicated. Detailed information regarding the type, timing, and duration of osteoporosis medication was not uniformly available throughout the retrospective study period.

### 2.4. Statistical Analysis

Continuous variables were analyzed using Student’s *t*-tests or Wilcoxon rank-sum test, depending on the normality of their distributions. Categorical variables were analyzed using the chi-square test or Fisher’s exact test, as appropriate. Postoperative TK, TL, and PJA were excluded because they could be directly affected by PJK. To evaluate the incremental value of postoperative FSPA beyond conventional postoperative PI-LL assessment, receiver operating characteristic (ROC) curves for postoperative FSPA and PI-LL were compared using the DeLong method. Nested logistic regression models were constructed by sequentially adding postoperative PI-LL and FSPA to a prespecified clinical model that included age, BMI, BMD, correction method, L5-S1 fusion method, and preoperative DLS. The adjusted associations of postoperative PI-LL and FSPA with PJK were estimated from the fully adjusted nested model. Improvements in model performance were assessed using likelihood-ratio (LR) tests, the Akaike information criterion (AIC), Nagelkerke R^2^, and the area under the ROC curve (AUC). Multicollinearity was assessed using variance inflation factors (VIFs), and model calibration was evaluated using the Hosmer-Lemeshow goodness-of-fit test. The FSPA threshold was determined using the Youden index. Interobserver reliability was assessed using all 258 postoperative radiographs, each independently measured by three observers. Intraclass correlation coefficients (ICCs) were calculated using a two-way random-effects, absolute-agreement model. Both single-measure reliability [ICC(2,1)] and average-measure reliability [ICC(2,3)] were calculated, with 95% confidence intervals obtained by bootstrap resampling with 10,000 iterations. The standard error of measurement (SEM) and 95% minimum detectable change (MDC95) were also calculated. Statistical significance was defined as *p* < 0.05.

## 3. Results

### 3.1. Baseline Characteristics

A total of 258 patients (mean age, 71.4 years; eight men and 250 women) met the inclusion criteria. The mean follow-up duration was 57.9 months. The mean BMI was 25.4 kg/m^2^, and BMD was 1.01 g/cm^2^. Pedicle subtraction osteotomy (PSO) was performed in 96 patients, whereas lateral interbody fusion (LLIF) with posterior column osteotomy (PCO) was performed in 162 patients. All patients underwent sacropelvic fixation.

### 3.2. Radiographic Parameters

Both groups had an average PI of 54.7° and 52.4°, respectively, without significant difference, which corroborates the high PI (>50°) reported by Roussouly et al. [22]. Baseline SVA was 205.2 mm in the non-PJK group and 205.9 mm in the PJK group, and baseline PI-LL mismatch was 58.3° in the non-PJK group and 55.7° in the PJK group, indicating severe sagittal malalignment. Baseline PT was 30.5° in the non-PJK group and 28.7° in the PJK group, indicating severe baseline PT for pelvic compensation (Table 1).

After deformity correction, a mean LL correction of 74.1° and 75.1°, respectively, was achieved without significant difference, but postoperative PI-LL was significantly less negative in the non-PJK group (−15.8° vs. −19.4°, *p* = 0.007). SVA and PT improved after surgery and at the final follow-up without significant difference between the two groups.

Preoperative SS did not differ significantly between groups (24.2° vs. 23.7°, *p* = 0.758), whereas the postoperative (48.2° vs. 45.3°, *p* = 0.041) and final follow-up (45.7° vs. 41.9°, *p* = 0.001) SS were significantly higher in the non-PJK group. Postoperative apex-LDI was higher in the non-PJK group (68.7% vs. 63.8%, *p* = 0.005). While the LS showed more lordosis preoperatively (−2.7° vs. −11.9°), immediately postoperatively (−27.5° vs. −30.5°), and at the final follow-up (−26.6° vs. −30.5°) in the PJK group, the degree of LS correction was greater in the non-PJK group (24.8° vs. 18.6°, *p* = 0.001). In addition, the PJK group demonstrated a higher postoperative LDI (43% vs. 39.3%, *p* = 0.012) and lower postoperative FSPA (−0.3° vs. 4.9°, *p* < 0.05). FSPA demonstrated excellent interobserver reliability: ICC(2,1) was 0.999 (95% CI, 0.9984–0.9993), and ICC(2,3) was 0.9997 (95% CI, 0.9995–0.9998). The SEM was 0.25°, and the MDC95 was 0.70°.

### 3.3. Factors Associated with PJK

There were no significant group differences in patient factors. Regarding surgical factors, LLIF with PCO (72.4%, *p* = 0.003) and PLIF at L5-S1 (63.4%, *p* = 0.024) were more frequently performed in the PJK group (Table 2). In the fully adjusted nested model (Table 3), FSPA remained independently associated with PJK (adjusted OR per degree, 0.910; 95% CI, 0.866–0.957; *p* < 0.001), whereas postoperative PI-LL was not significant (adjusted OR, 1.005; 95% CI, 0.974–1.038; *p* = 0.733). Adding FSPA to the clinical model containing postoperative PI-LL significantly improved model fit (LR χ^2^ = 15.11, *p* < 0.001), whereas adding postoperative PI-LL to the FSPA-containing model did not (LR χ^2^ = 0.12, *p* = 0.732) (Table 4).

### 3.4. ROC Analysis and FSPA Threshold Associated with PJK

**Figure 2:**
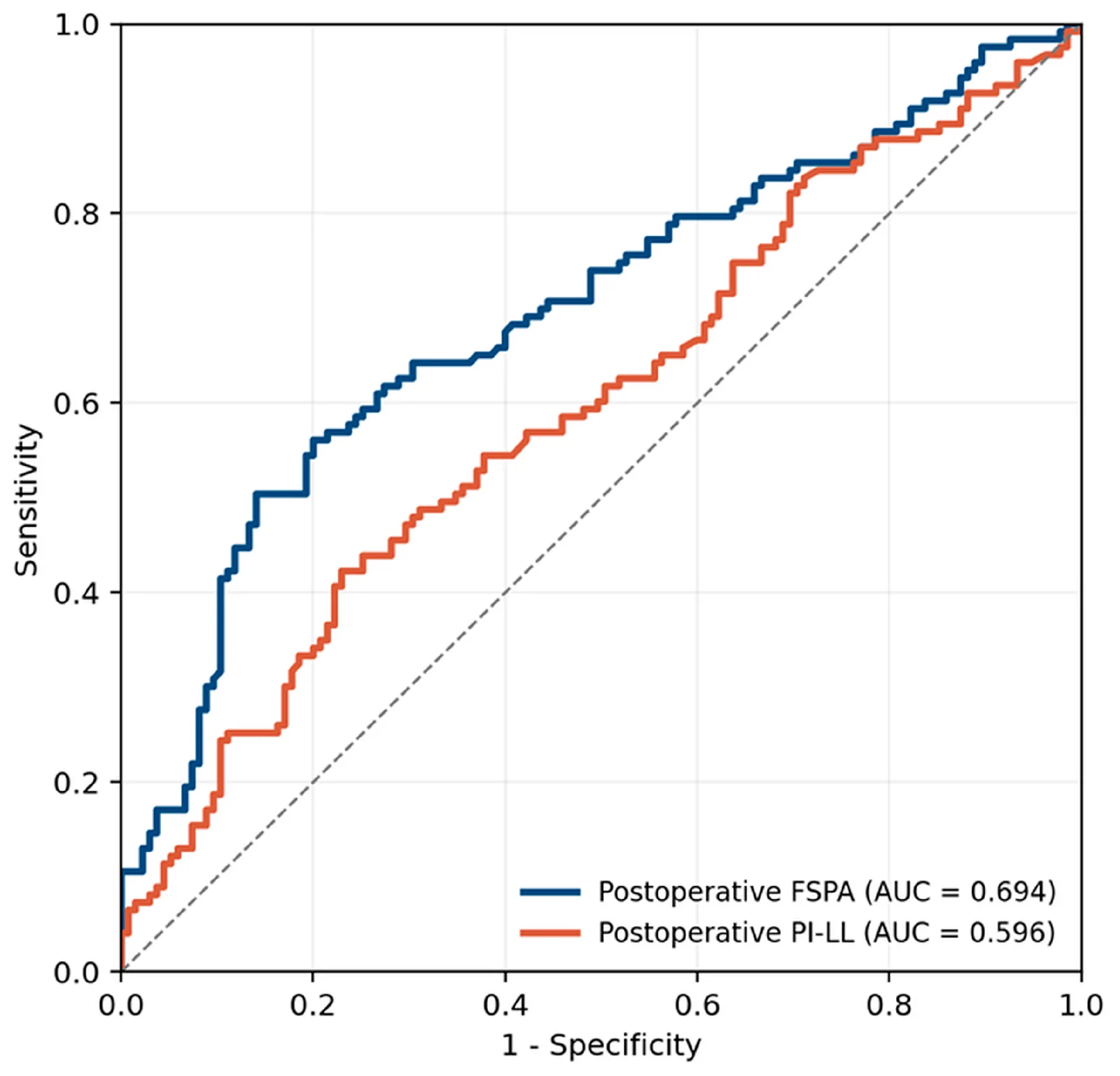
Comparative receiver operating characteristic curves for radiographic PJK. Postoperative FSPA demonstrated greater discriminatory performance than postoperative PI-LL (AUC, 0.694 vs. 0.596; difference, 0.099; 95% CI, 0.035–0.162; DeLong *p* = 0.002).

ROC analysis demonstrated moderate discriminatory ability of postoperative FSPA for PJK (AUC, 0.694; 95% CI, 0.629–0.760; *p* < 0.001), compared with an AUC of 0.596 (95% CI, 0.526–0.665) for postoperative PI-LL. FSPA had significantly greater discriminatory performance than postoperative PI-LL (AUC difference, 0.099; 95% CI, 0.035–0.162; DeLong *p* = 0.002) (Figure 2). The cohort-derived FSPA threshold was 2.38°, corresponding to a sensitivity of 64.2% and specificity of 63.7%. The positive predictive value was 61.7%, and the negative predictive value was 66.2%.

### 3.5. Correlation Between FSPA and Spinopelvic Parameters

Based on the correlation analysis (Table 5), FSPA had positive relationships with PI (*r* = 0.608), postoperative PI-LL (*r* = 0.610), and postoperative apex LDI (*r* = 0.266), and a negative correlation with postoperative LDI (*r* = −0.358). The degree of LL correction and LS correction was not correlated with the FSPA.

### 3.6. Relationship Between FSPA and Postoperative PI-LL

Linear regression (Table 6) demonstrated a significant relationship between FSPA and postoperative PI-LL (*r* = 0.61), yielding the equation: postoperative PI-LL = −19.546 + 0.826 × FSPA. For descriptive purposes, substituting the cohort-derived FSPA threshold of 2.38° into this equation yielded an estimated postoperative PI-LL of −17.6°. This estimate was not derived directly from PJK outcomes and should not be interpreted as an independent PI-LL threshold or surgical target.

## 4. Discussion

The primary goal of deformity correction surgery in ASD is to restore sagittal balance and achieve a physiological spinal alignment. Various mathematical prediction formulas and surgical guidelines have been introduced to restore and maintain sagittal balance [23,24,25], and further studies analyzing the ideal lordosis morphology and the relationship between lower and upper lumbar lordosis are being performed [19,22]. For the deformity correction, PI, a constant morphologic parameter, is the main guideline for surgery for ASD [17]. However, PI may not be completely invariant after long fusion. Cecchinato et al. [26] reported clinically relevant acute changes in PI following long fusion to S1, particularly in association with pelvic fixation. This result provides an additional perspective on the limitations of relying exclusively on PI-based alignment assessment and supports consideration of the achieved fused-construct geometry as a complementary measure.

In the present study, we specifically focused on patients with LDK, redefined as DBS [15], a distinct clinical entity characterized by pure sagittal malalignment associated with severe paraspinal muscle atrophy. In this context, “pure sagittal malalignment” refers to the predominance of sagittal deformity as the primary pathologic condition and surgical target, rather than the complete absence of concomitant degenerative coronal changes. LDK differs from other forms of ASD, such as postsurgical fixed sagittal deformity or degenerative/adult idiopathic scoliosis, in terms of pathophysiology and biomechanical characteristics. It is particularly prevalent in elderly women and has historically been associated with long-term squatting postures and floor-based lifestyles, which may contribute to progressive lumbar extensor muscle weakness and spinal degeneration [3,27,28,29,30].

**Figure 3:**
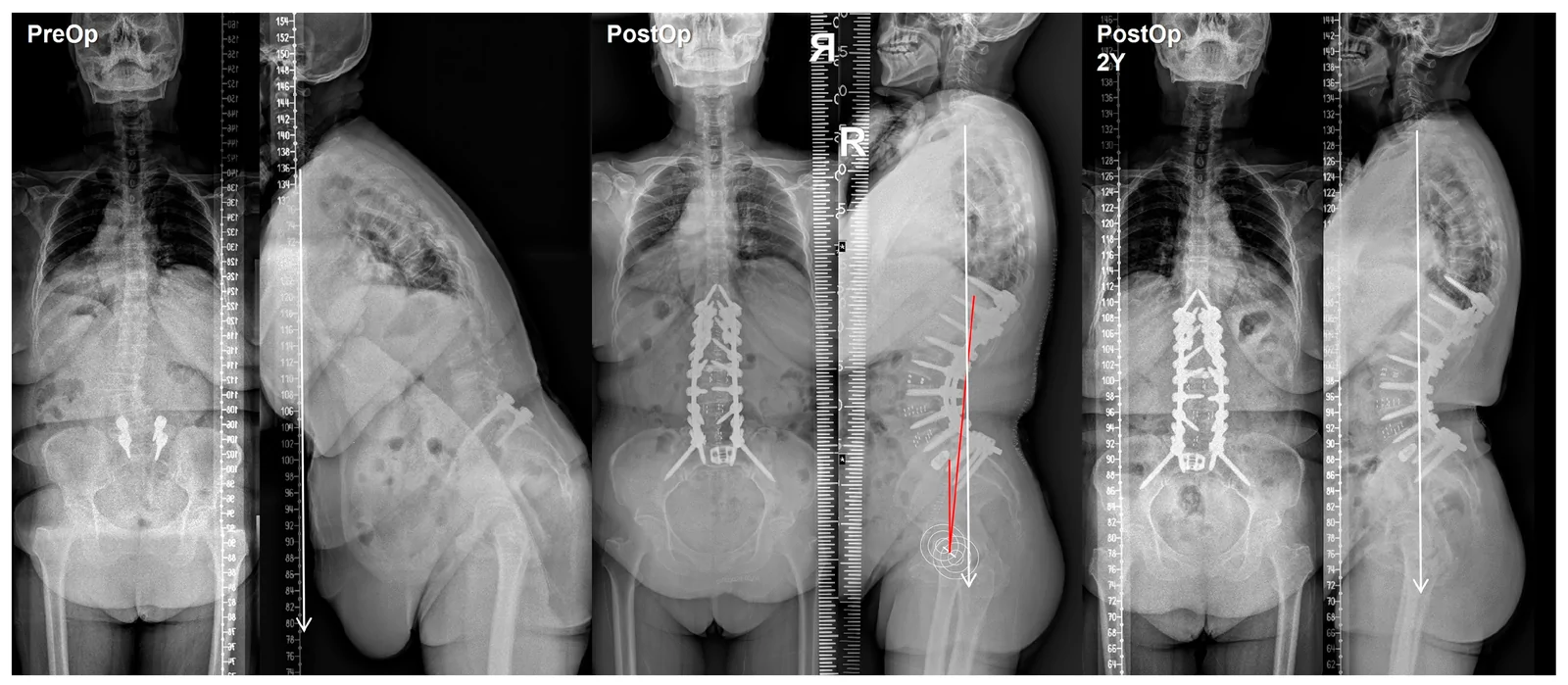
Representative case of proximal junctional kyphosis after overcorrection. A 69-year-old female with severe sagittal malalignment underwent long-segment fixation with ALIF at L5-S1 and LLIF with PCO at L2-5. Postoperatively, excessive correction resulted in a low FSPA (−5.7°) and PI-LL mismatch of −31°, followed by proximal junctional kyphosis with upper instrumented vertebra fracture. At 2-year follow-up, progression of PJK and compensatory increase in thoracic kyphosis were observed.

Yagi et al. [15] reported that although patients with DBS achieved initial postoperative restoration of sagittal balance comparable to those without DBS, they demonstrated greater loss of global sagittal alignment and a higher incidence of mechanical complications at 2-year follow-up (Figure 3). Similarly, Lee et al. [31] reported that patients with LDK tend to exhibit relatively high PI, low SS and LL, and elevated PT, consistent with the high pelvic incidence (>50°) reported by Roussouly et al. [22]. Our findings are in agreement with these studies, as both groups demonstrated high PI values (>50°) and markedly elevated preoperative PT, reflecting severe baseline pelvic compensation.

These characteristics suggest that conventional mathematical prediction formulas and guidelines may not fully capture the correction requirements of elderly patients with severe baseline sagittal malalignment. Recently, Passias et al. [4] introduced the concept of the “pelvic nonresponder”, referring to patients with high preoperative PI and PT who exhibit inferior clinical outcomes due to residual pelvic compensation despite adequate lordosis correction according to previously known formulas. They advocated for more aggressive lordosis correction, an overcorrection, in patients with severe baseline deformity. The cohort in our study closely resembles this “pelvic nonresponder” population [4], and numerous studies have reported improved clinical and radiographic outcomes with overcorrection strategies in this subgroup [28,32]. However, the appropriate degree of correction remains uncertain and may vary according to individual spinopelvic morphology and the severity of preoperative sagittal malalignment.

In this homogeneous cohort, postoperative FSPA remained independently associated with radiographic PJK after adjustment for postoperative PI-LL and relevant clinical and surgical variables. FSPA also demonstrated significantly greater discrimination than postoperative PI-LL, and adding FSPA improved the PI-LL-containing model, whereas adding PI-LL to the FSPA-containing model did not. These findings suggest that FSPA captures information regarding fused-construct orientation that is not fully represented by the magnitude of LL relative to PI. The 2.38° cutoff should nevertheless be considered a cohort-derived exploratory reference rather than a validated surgical target.

**Figure 4:**
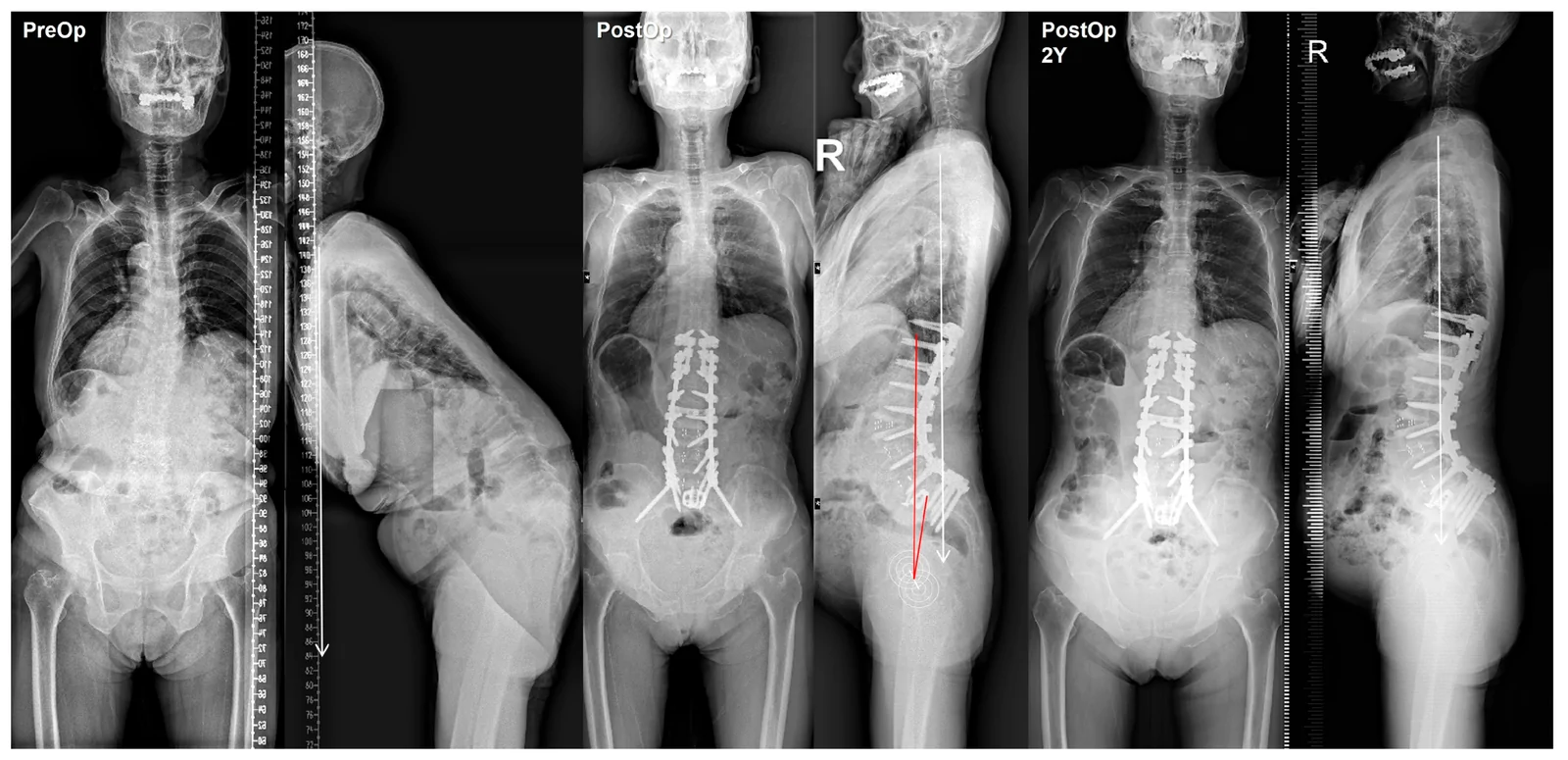
Representative case without proximal junctional kyphosis. A 73-year-old female with severe sagittal malalignment underwent long-segment fixation with ALIF at L5-S1 and LLIF with PCO at L2-5. Postoperatively, FSPA was 6.9° and PI-LL mismatch was −16°, achieving balanced sagittal alignment. At 2-year follow-up, alignment was maintained without development of proximal junctional kyphosis.

Because the fused spinal segments from the UIV to the pelvis can only move within the hip joint, FSPA can be considered a relatively position-independent angular parameter reflecting the geometric relationship between the UIV and pelvis. In this respect, FSPA shares conceptual advantages of angular parameters such as the T1PA and the L1PA [13,14], which integrate spinal inclination and pelvic orientation into a single value. Rather than simply quantifying the magnitude of LL relative to PI, FSPA may provide complementary information regarding the overall orientation of the surgically reconstructed spine relative to the pelvis. In addition, another potential advantage of FSPA is its intraoperative applicability. Previous studies have demonstrated that position-independent spinopelvic and vertebral-pelvic angular parameters can be assessed on intraoperative lateral imaging [33,34]. FSPA may similarly allow assessment of the achieved fused-construct alignment during surgery, providing immediate feedback regarding the extent of correction. This potential intraoperative applicability may enhance the practical value of FSPA in deformity correction. Therefore, the cohort-derived FSPA threshold of 2.38° and its corresponding estimated postoperative PI-LL of −17.6° may provide useful reference points for elderly patients with severe baseline deformity (Figure 4). Importantly, these values should be considered within a broader and individualized correction strategy rather than as isolated surgical targets. Deformity correction for ASD remains multifactorial and should incorporate patient demographics, bone quality, muscle status, pelvic morphology, global sagittal alignment, and the distribution of LL. Within this framework, FSPA may complement conventional alignment parameters by providing information on the orientation of the fused construct relative to the pelvis.

Nevertheless, these findings require cautious interpretation. The AUC of 0.694 and sensitivity and specificity of approximately 64% indicate only moderate discriminatory ability, and the threshold of 2.38° was derived and tested in the same cohort. Although FSPA added information beyond postoperative PI-LL in nested models, this does not establish causal or clinical utility. The estimated PI-LL value of −17.6° was obtained only by substitution into a regression equation between correlated postoperative measurements and was not outcome-derived. It is therefore reported descriptively and should not be regarded as an independent PI-LL threshold. External validation in more heterogeneous ASD populations is required.

Interestingly, the values of LDI using lower lumbar lordosis (L4-S1) and apex-LDI using lower lordosis arc angle showed different results when comparing non-PJK and PJK groups. Postoperative apex-LDI was higher in the non-PJK group, whereas postoperative LDI was higher in the PJK group. This discrepancy may be attributed to the relatively high PI and proximally located lumbar apex characteristic of patients with LDK/DBS in this study. Specifically, the postoperative LDI of 39.3% in the non-PJK group, which was below the GAP-recommended range of 50–80% [8], emphasizes the importance of PI-based lordosis morphology correction rather than simple quantitative lordosis correction by simply dividing the lower lumbar lordosis into L4-S1. Although further biomechanical studies are needed for clarification, to avoid PJK and obtain better clinical outcomes in high PI patients, surgeons may not only consider FSPA, but also aim to restore the total lumbar lordosis rather than solely focusing on lower lumbar lordosis correction, considering that the apex is rather higher in such patients.

### Limitations

This study had several limitations. Its retrospective, single-institution, single-surgeon design introduces potential selection bias and limits generalizability. The cohort consisted predominantly of elderly women with LDK/DBS who underwent a uniform T10-to-pelvis construct, limiting the generalizability of the findings to younger populations, men, other ASD etiologies, different UIV levels, low PI patients, and revision patients. The exact timing of PJK onset was not systematically available, precluding landmark or time-to-event analyses. Detailed measures of frailty, paraspinal muscle degeneration, and longitudinal osteoporosis treatment were not consistently available. Two GE Lunar DXA scanner models were used during the extended study period, and information regarding cross-calibration was not uniformly available, potentially introducing BMD measurement variability. Formal intraobserver reliability was not assessed. The present cohort included 149 patients who had also been included in our previous publication introducing the FSPA concept [12], corresponding to 78.4% of the previous cohort and 57.8% of the current cohort. However, all radiographic measurements and statistical analyses were newly performed for the present study, which addressed a distinct research question focusing on the discriminatory performance and incremental value of FSPA relative to postoperative PI-LL. Finally, the primary outcome was radiographic PJK; proximal junctional failure, revision surgery, patient-reported outcomes, and postoperative rehabilitation were not specifically evaluated. Further studies should extend beyond radiographic outcomes with a broader assessment of clinical benefit [35], and prospective multicenter studies with internal and external validation are required. Nevertheless, this study included a relatively large and homogeneous cohort of 258 patients with a single etiology who showed severe sagittal malalignment. The consistency in surgical technique and patient characteristics may reduce confounding factors and strengthen the reliability of the findings.

## 5. Conclusions

In this selected cohort of elderly patients with severe sagittal malalignment, higher postoperative FSPA was independently associated with lower odds of radiographic PJK. By reflecting the relationship between the fused spinal alignment and the pelvis, FSPA provided better discrimination and additional information beyond postoperative PI-LL. The identified threshold of 2.38° may offer a preliminary reference for postoperative alignment assessment in this population, although independent validation is warranted before its use as a surgical target.

## Figures and Tables

**Table 1:** Comparison of radiographic and clinical characteristics between the Non-PJK and PJK groups.

Variables	Non-PJK (*n* = 135)	PJK (*n* = 123)	*p*-Value
Age at surgery (years)	71 ± 5.5	71.9 ± 5.4	0.217
BMI (kg/m^2^)	25.3 ± 3.5	25.5 ± 3.2	0.592
BMD (g/cm^2^)	1 ± 0.22	1.02 ± 0.22	0.501
Pelvic incidence (PI, °)	54.7 ± 10.5	52.4 ± 11.7	0.101
Sacral slope (°)			
preoperation	24.2 ± 11.7	23.7 ± 12.8	0.758
postoperation	48.2 ± 12	45.3 ± 10.4	0.041 *
last follow-up	45.7 ± 8.7	41.9 ± 8.7	0.001 *
Pelvic tilt (°)			
preoperation	30.5 ± 12.7	28.7 ± 12.2	0.249
postoperation	6.5 ± 11.6	7.1 ± 11.1	0.667
last follow-up	9 ± 9.5	10.5 ± 9.7	0.206
Sagittal vertical axis (SVA, mm)			
preoperation	205.2 ± 64.7	205.9 ± 50.6	0.914
postoperation	−13.7 ± 30.5	−11.8 ± 39.7	0.677
SVA correction	218.8 ± 71	217.8 ± 63.5	0.901
last follow-up	−0.1 ± 31	4.6 ± 36.7	0.268
SVA loss	13.6 ± 35.9	16.5 ± 48.7	0.590
Thoracic kyphosis (°)			
preoperation	1.1 ± 19.1	12.1 ± 15.5	<0.001 *
postoperation	22 ± 10.5	36.3 ± 12.1	<0.001 *
last follow-up	29.2 ± 11.2	47.1 ± 11.5	<0.001 *
Thoracolumbar junctional angle (°)			
preoperation	3.8 ± 15.9	14.7 ± 17.7	<0.001 *
postoperation	−12.2 ± 18.3	−1.6 ± 17.3	<0.001 *
last follow-up	−7.3 ± 19	3.4 ± 17.7	<0.001 *
Lumbar lordosis (LL, °)			
preoperation	3.6 ± 18.1	3.3 ± 18.1	0.878
postoperation	−70.5 ± 10.7	−71.8 ± 9.8	0.304
LL correction	74.1 ± 17.8	75.1 ± 17.4	0.656
last follow-up	−66.9 ± 18.8	−69.2 ± 17.3	0.294
LL change	3.6 ± 15.6	2.6 ± 15.6	0.591
Lumbosacral junctional angle (°)			
preoperation	−2.7 ± 13.4	−11.9 ± 15.6	<0.001 *
postoperation	−27.5 ± 9.5	−30.5 ± 9.9	0.014 *
last follow-up	−26.6 ± 10.9	−30.5 ± 9.7	0.002 *
PI-LL (°)			
preoperation	58.3 ± 18.4	55.7 ± 16.7	0.233
postoperation	−15.8 ± 10.4	−19.4 ± 11.2	0.007 *
last follow-up	−12.2 ± 18.6	−16.8 ± 18.8	0.047 *
Proximal junctional angle (°)			
preoperation	−2 ± 6.3	1.1 ± 5.4	<0.001 *
postoperation	6.6 ± 4.5	17 ± 8.2	<0.001 *
last follow-up	9.7 ± 4.7	25.6 ± 10.5	<0.001 *
FSPA (°)	4.9 ± 7.1	−0.3 ± 7.9	<0.001 *
Postoperative apex-LDI (%)	68.7 ± 15.2	63.8 ± 12.1	0.005 *
Last apex-LDI (%)	64.7 ± 19.4	58.4 ± 14.3	0.003 *
Postoperative LDI (%)	39.3 ± 12.4	43 ± 13.1	0.012 *
Last LDI (%)	39 ± 14.1	42.5 ± 15.4	0.055

Data are presented as mean ± standard deviation. * *p* < 0.05. BMD, bone mineral density; BMI, body mass index; Last, last follow-up; FSPA, fused spinopelvic angle; LDI, lordosis distribution index.

**Table 2:** Comparison of categorical variables between the Non-PJK and PJK groups.

Variables	Non-PJK (*n* = 135)	PJK (*n* = 123)	*p*-Value
Rod fracture (RF)			
Non-RF	107 (79.3%)	111 (90.2%)	0.016 *
RF	28 (20.7%)	12 (9.8%)	
Accessory rod technique (AR)			
Non-AR	85 (63%)	64 (52%)	0.079
AR	50 (37%)	59 (48%)	
Correction method			
LLIF	73 (54.1%)	89 (72.4%)	0.003 *
PSO	62 (45.9%)	34 (27.6%)	
Preoperative degenerative lumbar scoliosis (DLS)			
No DLS	87 (64.4%)	91 (74%)	0.107
DLS	48 (35.6%)	32 (26%)	
L5-S1 fusion method			
PLIF	66 (48.9%)	78 (63.4%)	0.024 *
ALIF	69 (51.1%)	45 (36.6%)	

* *p* < 0.05. PSO, pedicle subtraction osteotomy; LLIF, lateral lumbar interbody fusion; PLIF, posterior lumbar interbody fusion; ALIF, anterior lumbar interbody fusion.

**Table 3:** Postoperative alignment parameters in the fully adjusted logistic regression model for PJK.

Variables	RC	SE	Adjusted OR	95% CI	*p*-Value
Post PI-LL, per degree	0.0055	0.0160	1.005	0.974–1.038	0.733
Post FSPA, per degree	−0.0938	0.0252	0.910	0.866–0.957	<0.001 *

The model was adjusted for age, BMI, BMD, correction method, L5-S1 fusion method, and preoperative DLS. * *p* < 0.05. RC, regression coefficient; SE, standard error; CI, confidence interval; FSPA, fused spinopelvic angle; PI, pelvic incidence; LL, lumbar lordosis; BMI, body mass index; BMD, bone mineral density; DLS, degenerative spondylosis.

**Table 4:** Incremental predictive value of postoperative FSPA beyond postoperative PI-LL in nested logistic regression models for PJK.

Model	AUC	AIC	Nagelkerke R^2^	Incremental Test
Clinical model	0.631	355.70	0.077	Reference
Clinical + postoperative PI-LL	0.657	351.51	0.107	Vs. clinical : LR χ^2^ = 6.18, *p* = 0.013
Clinical + FSPA	0.715	336.52	0.176	Vs. clinical : LR χ^2^ = 21.18, *p* < 0.001
Clinical + postoperative PI-LL + FSPA	0.717	338.40	0.177	FSPA added to PI-LL model : LR χ^2^ = 15.11, *p* <0.001; PI-LL added to FSPA model : LR χ^2^ = 0.12, *p* = 0.732

The clinical model included age, BMI, BMD, correction method, L5-S1 fusion method, and preoperative DLS. AUC, area under the receiver operating characteristic curve; AIC, Akaike information criterion; FSPA, fused spinopelvic angle; PI, pelvic incidence; LL, lumbar lordosis; LR, likelihood ratio; BMI, body mass index; BMD, bone mineral density; DLS, degenerative lumbar scoliosis.

**Table 5:** Correlations between FSPA and radiologic parameters.

	PI	PO SS	PO LS	LS cor	PO LL	LL cor	PO PI-LL	PO Apex- LDI	PO LDI
FSPA	0.608 **	0.212 **	0.325 **	0.019	−0.032	−0.088	0.610 **	0.266 **	−0.358 **

** *p* < 0.01. PO, postoperative; PI, pelvic incidence; SS, sacral slope; LS, lumbosacral junctional angle; cor, correction; LL, lumbar lordosis; LDI, lordosis distribution index; FSPA, fused spinopelvic angle.

**Table 6:** Linear regression analysis of postoperative PI-LL according to FSPA.

	Unstandardized Coefficients		Standardized Coefficients β			
	B	SE		t	Significance	95% CI
FSPA	0.826	0.067	0.611	12.345	<0.001 *	0.694, 0.958
Constant	−19.546	0.556		−35.151	<0.001	−20.652, −18.456

R^2^ = 0.373. * *p* < 0.05. PI, pelvic incidence; LL, lumbar lordosis; SE, standard error; FSPA, fused spinopelvic angle.

## Data Availability

The data presented in this study are available on request from the corresponding author. The data are not publicly available because participant consent for public data sharing was not obtained.

## Abbreviations

The following abbreviations are used in this manuscript: ASD, adult spinal deformity; PJK, proximal junctional kyphosis; UIV, uppermost instrumented vertebra; FSPA, fused spinopelvic angle; PI, pelvic incidence; T1PA, T1 pelvic angle; L1PA, L1 pelvic angle; LDK, lumbar degenerative kyphosis; DBS, drop body syndrome; SVA, sagittal vertical axis; LL, lumbar lordosis; PT, pelvic tilt; CT, computed tomography; SS, sacral slope; TK, thoracic kyphosis; TL, thoracolumbar junctional angle; LS, lumbosacral junctional angle; LDI, lordosis distribution index; PJA, proximal junctional angle; BMD, bone mineral density; BMI, body mass index; DLS, degenerative lumbar scoliosis; ALIF, anterior lumbar interbody fusion; PLIF, posterior lumbar interbody fusion; ROC, receiver operating characteristic; PSO, pedicle subtraction osteotomy; LLIF, lateral interbody fusion; PCO, posterior column osteotomy.
